# Supplementary figures and images for: S100A4 inhibits cell proliferation by interfering with the S100A1-RAGE V domain
Source: PLoS One. 2019 Feb 19;14(2):e0212299. doi: 10.1371/journal.pone.0212299 (PMC6380570; doi:10.1371/journal.pone.0212299)

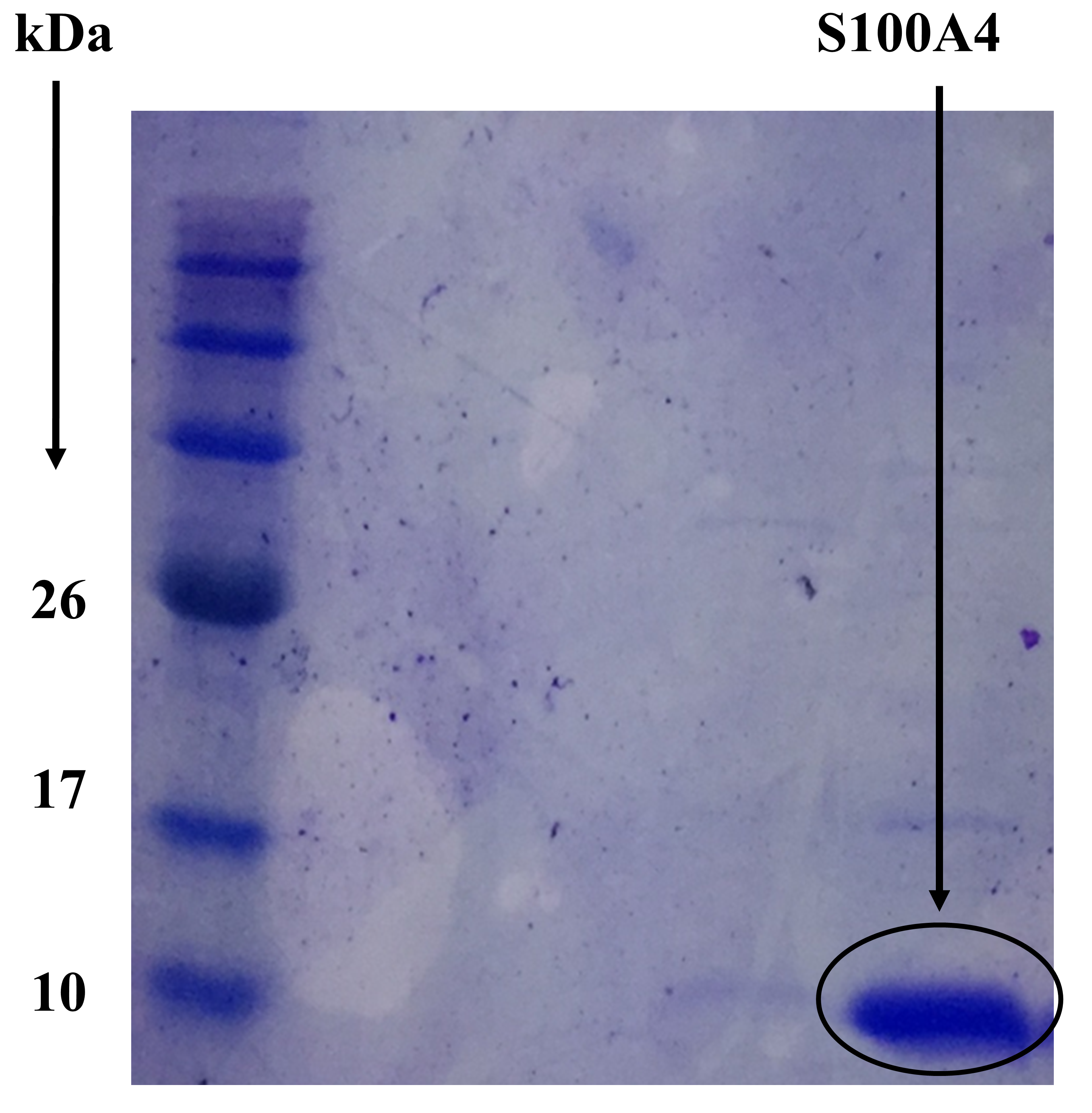

Supplement: S1 Fig — (TIF) [file pone.0212299.s001.tif]

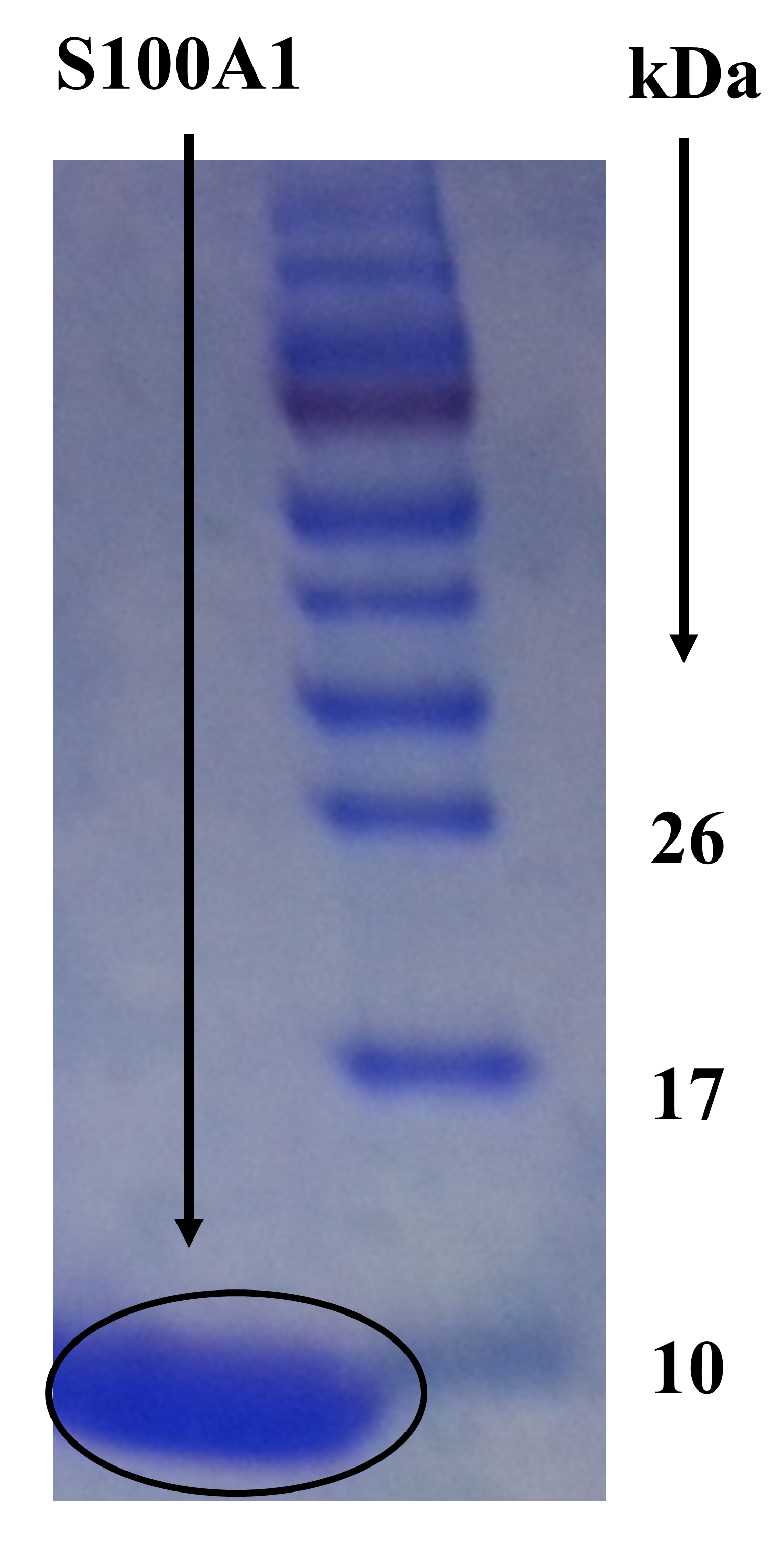

Supplement: S2 Fig — (TIF) [file pone.0212299.s002.tif]

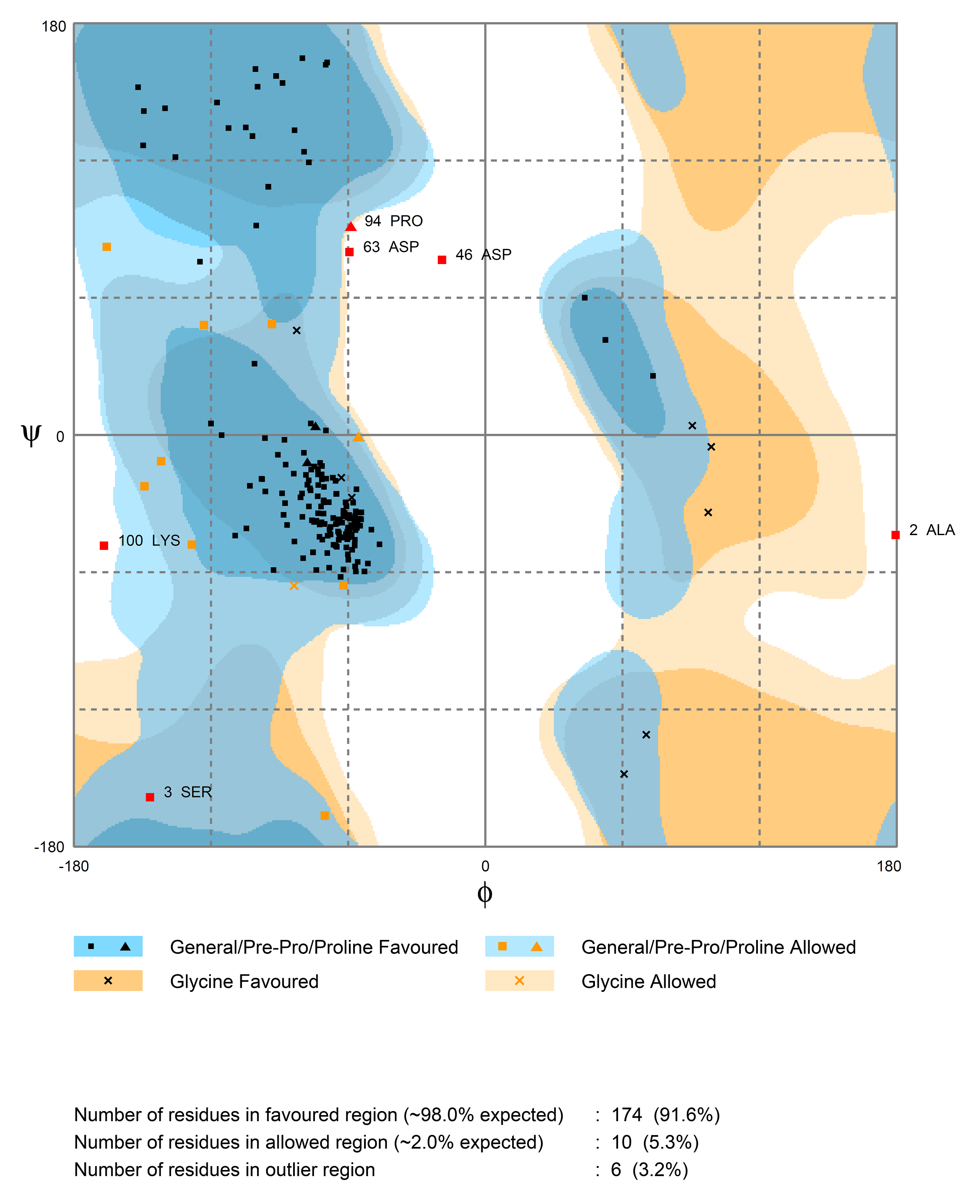

Supplement: S3 Fig — Ninety-one percent of the residues were in the favored area, 5.3% were in the allowed area, and 3.2% were in the disallowed region. (TIF) [file pone.0212299.s003.tif]
